# Phenotyping neuroblastoma cells through intelligent scrutiny of stain-free biomarkers in holographic flow cytometry

**DOI:** 10.1063/5.0159399

**Published:** 2023-09-21

**Authors:** Daniele Pirone, Annalaura Montella, Daniele Sirico, Martina Mugnano, Danila Del Giudice, Ivana Kurelac, Matilde Tirelli, Achille Iolascon, Vittorio Bianco, Pasquale Memmolo, Mario Capasso, Lisa Miccio, Pietro Ferraro

**Affiliations:** 1 CNR-ISASI, Institute of Applied Sciences and Intelligent Systems “E. Caianiello,” via Campi Flegrei 34, 80078 Pozzuoli, Napoli, Italy; 2 Department of Electrical Engineering and Information Technologies (DIETI), Università degli Studi di Napoli “Federico II,” via Claudio 21, 80125 Napoli, Italy; 3 CEINGE Biotecnologie Avanzate, Napoli, Italy; 4 Department of Molecular Medicine and Medical Biotechnology (DMMBM), Università degli Studi di Napoli “Federico II,” Napoli, Italy; 5 Department of Medical and Surgical Sciences (DIMEC) – Alma Mater Studiorum—University of Bologna, 40138 Bologna, Italy; 6 Centre for Applied Biomedical Research (CRBA), University of Bologna, 40138 Bologna, Italy; 7 European School of Molecular Medicine, Università degli Studi di Milano, Milano, Italy

## Abstract

To efficiently tackle certain tumor types, finding new biomarkers for rapid and complete phenotyping of cancer cells is highly demanded. This is especially the case for the most common pediatric solid tumor of the sympathetic nervous system, namely, neuroblastoma (NB). Liquid biopsy is in principle a very promising tool for this purpose, but usually enrichment and isolation of circulating tumor cells in such patients remain difficult due to the unavailability of universal NB cell-specific surface markers. Here, we show that rapid screening and phenotyping of NB cells through stain-free biomarkers supported by artificial intelligence is a viable route for liquid biopsy. We demonstrate the concept through a flow cytometry based on label-free holographic quantitative phase-contrast microscopy empowered by machine learning. In detail, we exploit a hierarchical decision scheme where at first level NB cells are classified from monocytes with 97.9% accuracy. Then we demonstrate that different phenotypes are discriminated within NB class. Indeed, for each cell classified as NB its belonging to one of four NB sub-populations (i.e., CHP212, SKNBE2, SHSY5Y, and SKNSH) is evaluated thus achieving accuracy in the range 73.6%–89.1%. The achieved results solve the realistic problem related to the identification circulating tumor cell, i.e., the possibility to recognize and detect tumor cells morphologically similar to blood cells, which is the core issue in liquid biopsy based on stain-free microscopy. The presented approach operates at lab-on-chip scale and emulates real-world scenarios, thus representing a future route for liquid biopsy by exploiting intelligent biomedical imaging.

## INTRODUCTION

Neuroblastoma (NB), the most common pediatric solid tumor of the sympathetic nervous system, represents a biological and clinical heterogeneous cancer that ranges from tendency for spontaneous regression to a highly aggressive metastasized tumor phenotype that could be unresponsive to standard treatment.^1^ This malignancy, which develops anywhere along the sympathetic chain, exhibits early age of onset, with a median age at the diagnosis of about 19 months and accounts for approximately 15% of children cancer-related mortality.^1^ In the last few years, the identification of diverse genomic markers has contributed to the risk stratification and improvement of NB patients' survival rate.^2^ Indeed, several recurrent segmental chromosomal alterations have been demonstrated to discriminate between low-risk and high-risk patients.^2,3^ Additionally, genome-wide association studies, high-throughput sequencing, and microarray gene expression-based studies have identified multiple genetic changes that characterize NB both hereditable and somatically acquired^4–6^ and that are promising prognostic predictors and therapeutic targets. Genetic alterations occurring in non-coding DNA such as TERT rearrangements^7^ and point mutations in regulatory elements of transcription factor binding sites^8,9^ also contribute to NB development. Despite these advances in genomic research, treatment of NB is still unsuccessful in half of the patients diagnosed with the high-risk form. To date, for NB diagnosis and monitoring, tumor biopsy followed by serial imaging scans and blood and urine catecholamine tests are used.^10^ Although tissue biopsy is considered to be the gold standard for biomarkers identification for personalized medicine, it seems to be unable to capture the complexity underlying NB heterogeneity. Moreover, besides the limitations linked to costs and risks for patients, this test is representative only of the sampling site and most importantly it does not allow the monitoring of cancer progression and therapy adjustment.^11^ Therefore, in pediatric patients, the risks linked to tissue biopsy sometimes may exceed benefits.

Over the last few years, the sampling and analysis of non-solid biological tissue (e.g., blood) named “liquid biopsy” (LB) has been aimed to overcome these limitations. Peripheral blood from cancer patients may contain tumor-derived and tumor-associated components.^12^ Recently, it has been showed that the deep targeted sequencing approach to identify tumor-specific alterations in cell-free tumor DNA can be a valid tool for improving diagnosis and monitoring disease progression.^13^ In addition to cell-free tumor DNA, circulating tumor cells (CTCs) represent a snapshot of overall tumor bulk (primary tumor and metastases).^14^ CTCs detach from the primary tumor and disseminate to distant sites via blood singularly or in clusters.^15^ Moreover, undergoing epithelial-to-mesenchymal transition (EMT), these cells lose cell contacts and acquire more motile and less differentiated phenotype.^16,17^ The identification of CTCs constitutes a well-validated and reproducible technology with several potential applications in early cancer diagnosis and prognosis, which may provide a direct measure of tumor spatial and temporal heterogeneity.^14^ Moreover, CTCs can be cultured *ex vivo* to perform single-cell analysis and functional assays (e.g., drug sensitivity assays) leading to discovery of new therapeutic targets or resistance mechanisms.^18,19^ However, because of their low representativeness within the peripheral blood (1–10 cells per 1 ml), isolation and efficient enrichment of these CTCs represent a great challenge to date. Current technologies for CTCs isolation and enumeration are label-dependent (affinity-based) methods, based on cell selection by using antibodies against cell-surface tumor antigens, such as adhesion molecules of epithelial cells (EpCAM) and cytokeratin (CK).^20^ Among them, CellSearch^®^ system, the only FDA-approved clinical application platform, selects CTCs expressing EpCAM and CK, which do not exhibit the leukocyte marker CD45 on their cell surface. Given label-dependent strategies limitations, label-free methods have been developed to improve the CTCs enrichment and isolation. To date, EpCAM-independent assay combined with immunostaining-fluorescence *in situ* hybridization has been used to CTCs detection and enrichment in NB patients, demonstrating that the number of these cells is significantly correlated with overall survival.^21^ Moreover, another approach based on the use of Amnis Image Stream Imaging Flow Cytometer, which combines flow cytometry with fluorescence microscopy, highlighted the clinical utility of CTCs as novel therapeutic biomarkers in high-risk NB patients under chemotherapy treatment.^22^ However, CTCs enrichment and isolation in NB patients remain challenging because of the unavailability of universal and specific cell-surface markers for NB cells. In this context, many advanced technologies combining microfluidic platforms with label-free imaging techniques and artificial intelligence (AI) may represent a useful tool to efficiently discriminate tumor cells from other cell types or within a background of blood cells.^23,24^

Digital holography (DH) has recently shown a great potential as optical microscopy technique. In fact, it allows collect the whole information (i.e., both the amplitude and phase) about a wavefront transmitted through a biological specimen. Such information takes the form of a 2D image by means of the light interference principles,^25^ thus avoiding any exogenous label to assure proper phase-contrast imaging. Most of the morphological information related to each cell is embedded into the quantitative phase map (QPM) retrieved from the recorded digital hologram.^26^ The possibility of measuring label-free quantitative features, e.g., dry mass and biovolume, related to the cell biophysical properties^27^ has made quantitative phase imaging (QPI) a powerful tool increasingly exploited in biomedicine and biomedical imaging.^28–32^ Moreover, DH has the unique capability of neglecting the time-consuming placement of the imaged sample in the correct focal plane before every acquisition, since it can be numerically refocused after the experiment.^33^ Also due to the simpler sample's preparation protocols, DH can be easily combined to flow cytometry,^34–36^ thus allowing for biomedical applications requiring the high-throughput property. Many applications based on DH in flow cytometry have been developed for the identification of cancer cells,^37–43^ thanks to its ability of quickly screening large volumes. They are mostly based on the AI, since DH in flow cytometry allows answering to its increasing demand for huge training datasets by collecting large amounts of single-cell quantitative information in very short times.^44^ Recently, it has been also demonstrated the possibility to classify healthy and cancer cell lines acquired by quantitative phase imaging exploiting a small training set, i.e., the TOP-GAN algorithm.^45^ Furthermore, the combination between holographic flow cytometry and AI has been also demonstrated fruitful in the environmental field. For example, deep learning has been exploited for the fast reconstruction of ocean samples' images recorded by a field-portable holographic flow cytometer,^46^ and the same system has been exploited for the deep learning-based label-free phenotyping of marine microalgae populations.^47^

Here, we show, for the first time, that phenotyping different sub-populations of the same type of cancer is possible by means of QPI operating in flow-cytometry modality. In fact, thanks to the combination of AI with a holographic microscope coupled to a microfluidic channel, we classify, as a first step of a hierarchical approach, NB cells from monocytes in label-free mode. This is a key issue, worth to be pointed out, as monocytes are the most similar white blood cells (WBCs) that can be failed to be filtered out by existing sorting technologies. The second task of the processing pipeline is remarkably more challenging to tackle. Following the hierarchical classification scheme, we developed a smart strategy to identify, with high degree of confidence and for each single cell judged as NB at the first step, the four different NB subtypes considered here, i.e., CHP212, SKNBE2, SHSY5Y, and SKNSH. We selected two cell lines with (CHP212 and SKNBE2) and without (SHSY5Y and SKNSH) *MYCN* amplified and 1p36 deletion, well-known genomic markers of unfavorable clinical outcome. The cell line SHSY5Y is a subline of SKNSH, thus having similar phenotypes, and these cell lines are a good control for assessing the classification ability of our proposed method. The more complex sample conceived herein in our investigation aims to emulate a case quite close to what might be found in a real-world liquid biopsy test. One of the key points that allowed us to reach the goal shown in this paper is linked to the quite common behavior of suspended cells flowing along a microfluidic channel. In fact, a profitable case for imaging purposes occurs when cells experience rotation while they flow inside the FOV. Rotation allows to collect a very large dataset of QPMs where the system offers different views of each cell. Hence, we can avoid the common paradigm of data augmentation, employed in all the conventional AI-based classification approaches, in which the rotation of images is implemented numerically. Tumbling of cells during flow along a microfluidic channel is well known and it allows to get also their full tomograms, provided that a uniform angular rotation around a fixed axis is assured.^48–53^ Recently, identification of tumor cells against white blood cells has been demonstrated by the AI-powered tomographic phase imaging flow cytometry system with a success rate higher than 97% in the recognition of tumor cells. Moreover, the system was able to distinguish by two different cancer cell types, i.e., NB and ovarian cancer, with accuracy over 97%.^54^ In the case considered here, the big advantage is that we do not need to meet such demanding experimental constrains, which leads to a considerable simplification of the opto-fluidic recording system. This allows collecting hundreds of angular digital holograms for each cell by a quite fast recording step.

To solve the classification problem, three image analysis concepts are introduced in this work, each of them providing a significant boost to the classification performance. (i) We build an *ad hoc* three-level hierarchical classifier fed by the thousands of collected QPMs; (ii) from the QPMs, we extract a new set of features based on the fractal geometry framework; and (iii) a max-voting strategy is implemented, exploiting the experimental conditions of the proposed system. The fractal features are shown to define a more distinctive fingerprint about the analyzed cell lines. Indeed, fractal geometry was introduced to provide a full insight of nature, more complete than the classical Euclidean one.^55^ The advantages of fractal geometry have been also demonstrated in biology and medicine,^56^ and recently, it has been applied to solve the challenging task of discerning microplastics from microalgae in water samples by machine learning and QPI.^57^ For the first time, here we show the advantageous use of fractal analysis to classify human circulating cells. As for the max-voting strategy, this is based on the physical data augmentation discussed above and is exploited here to reduce the random classification error obtained in case each cell is represented by one single QPM. The methodology proposed here opens a new path toward the realization of the liquid biopsy paradigm.^58^

## RESULTS

### Data collection and inspection

**FIG. 1.**
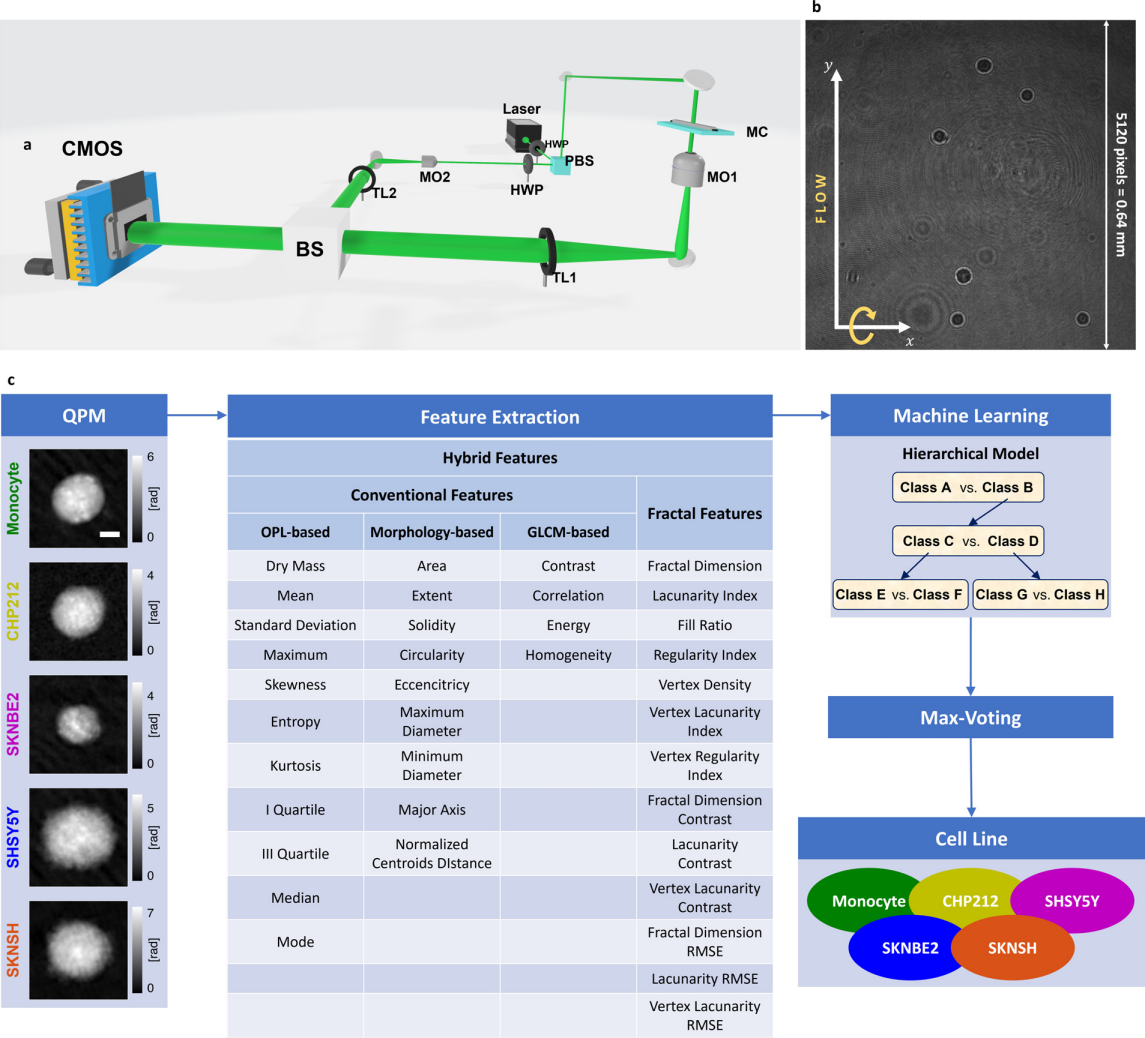
Holographic imaging flow cytometry for distinguishing several subtypes of NB cells (i.e., CHP212, SKNBE2, SHSY5Y, and SKNSH) from monocytes. (a) Sketch of the holographic imaging flow cytometer. HWP: half-wave plate; PBS: polarizing beam splitter; L1, L2: Lens; M: mirror; MO: microscope objective; MC: microfluidic channel; TL: tube lens; BS: beam splitter; CMOS: camera. (b) Digital hologram recorded by the holographic imaging flow cytometer, with cells imaged while flowing along the y-axis and rotating outside the image plane. (c) Overall pipeline of the proposed strategy. N QPMs ( 200×200 square pixels size, 5 *μ*m scale bar) for each cell are numerically retrieved from the recorded digital holograms, and 37 features are measure for each of them. The extracted features are fed to a hierarchical classifier (see the sketch with fake class names). The N predicted outputs are used to infer the cell line of the analyzed cell by means of max-voting.

To solve the problem of identifying the NB cells among the most similar WBCs, i.e., the monocytes, and to further distinguish NB cancer subtypes (i.e., CHP212, SKNBE2, SHSY5Y, and SKNSH), the holographic imaging flow cytometry system sketched in Fig. 1(a) has been employed to collect a suitable image dataset (see the description of the setup in the Methods section). In particular, for each cell, several digital holograms like that in Fig. 1(b) are acquired along a fixed beam direction at multiple viewing angles. Then, their corresponding QPMs are numerically retrieved, as shown in Fig. 1(c) (see the description of the holographic processing in the Methods section). For this reason, we collected 82 594 QPMs related to 563 cells, distributed among different cell lines as reported in Table I. A QPM is an image dense of quantitative information despite its 2D form. In fact, the phase values encoded inside a QPM can be interpreted as^27^

$$\left\{\begin{array}{l} QPM\left(x,y\right)=\frac{2\pi }{\lambda }OPL\left(x,y\right)\\ OPL\left(x,y\right)=\int_{z}\left[n\left(x,y,z\right)-n_{0}\right]dz, \end{array}\right.$$ (1)

where OPL is the optical path length, λ is the central wavelength, $n\left(x,y,z\right)$ is the 3D spatial distribution of the cell RI, $n_{0}$ is the RI of the surrounding medium, and z is the optical axis. Therefore, both the 3D morphology and the 3D RI spatial distribution of a cell are gathered inside its 2D QPM without the need for exogenous labels, unlike fluorescence imaging. Due to the possibility of measuring label-free quantitative features related to the cell biophysical properties,^28^ the QPMs have been exploited for detecting the NB cells and their subtypes. To this aim, we have followed the pipeline sketched in Fig. 1(c). After reconstructing the N QPMs of a flowing and rolling cell, 37 features are measured from each of them and are fed to a hierarchical machine learning classifier. The classes predicted for QPMs of a single cell are combined by means of a max-voting strategy in order to infer the cell line it belongs to.

**TABLE I.** Dataset collected by the Holographic imaging flow cytometer.

Cell line		No. of cells	No. of QPMs
Monocyte	THP-1	247	30 291
Neuroblastoma	CHP212	115	12 108
	SKNBE2	106	11 959
	SHSY5Y	66	9416
	SKNSH	151	18 820

**FIG. 2.**
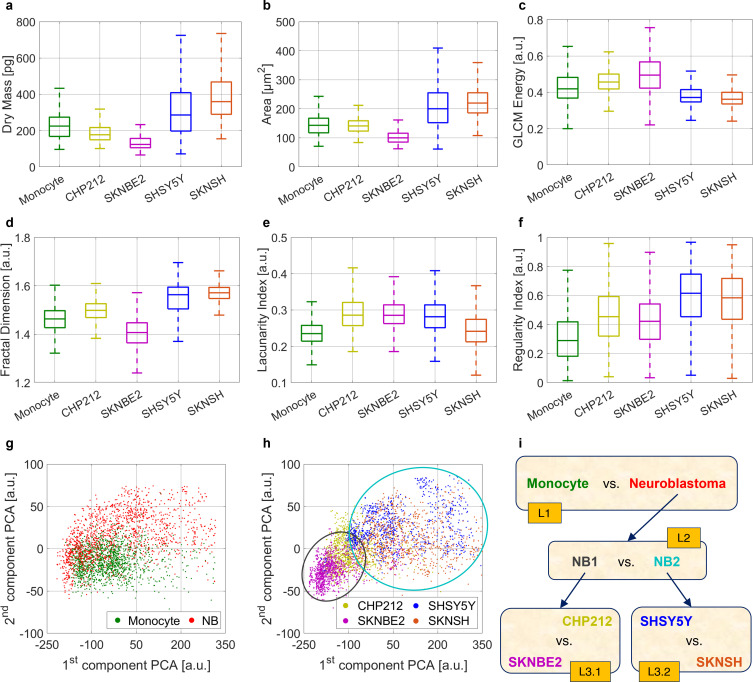
Inspection of the dataset collected by the holographic imaging flow cytometer. (a)–(c) Box plots about some conventional features, i.e., dry mass, area, and GLCM energy, respectively, computed from the QPMs for each single cell. (d)–(f) Box plots about some fractal features, i.e., fractal dimension, lacunarity index, and regularity index, respectively, computed from the QPMs for each single cell. (g) Scatter plot of the first two PCA components computed from all the conventional and fractal features about monocytes and NB cells. (h) Scatter plot of the first two PCA components computed from all the conventional and fractal features about the four NB subtypes. (i) Sketch of the hierarchical classifier made of three levels (L1, L2, and L3) and four single classifiers (L1, L2, L3.1, and L3.2). The intermediate NB1 class includes CHP212 and SKNBE2 cells [gray circle in (h)]. The intermediate NB2 class includes SHSY5Y and SKNSH cells [cyan circle in (h)].

In particular, for each QPM, we started from computing a set of 24 features [see the 24 features listed in the first three columns of the table in Fig. 1(c) and the corresponding description in the Methods section], selected among those usually measured in QPI-based machine learning problems and termed here conventional features. Among them, 11 features are strictly related to the OPL, 9 features derive from the cell 2D morphology, and the remaining 4 features are based on the Gray-Level Co-occurrence Matrix (GLCM). In Figs. 2(a)–2(c), we report the histograms of the dry mass (OPL-based, defined as the amount of non-aqueous content inside the cell^27^) the area (morphology-based), and the energy (GLCM-based), measured on monocytes and NB cells.

In addition, we have considered a set of other 13 features, computed by applying the principles of fractal geometry [see the 13 features listed in the last column of the table in Fig. 1(c) and the corresponding description in the Methods section]^57^ and termed here fractal features. The histograms of some of these, i.e., the fractal dimension, the lacunarity index, and the regularity index, measured on monocytes and NB cells, are displayed in Figs. 2(d)–2(f).

We further deepened the data investigation by carrying out the principal component analysis (PCA) over the whole dataset, i.e., considering both conventional and fractal features. In Fig. 2(g), the first two PCA components are shown by highlighting the monocytes vs all the NB cells, while in Fig. 2(h), the first two PCA components of the four NB subtypes are represented. The existence of two distinct groupings inside the NB family is evident in Fig. 2(h), i.e., CHP212+SKNBE2 and SHSY5Y+SKNSH, as indicated by the circles. This is quantitatively confirmed by the distance between all the possible groups of two classes computed by means of the Fisher's criterion^59^ over the first two PCA components, that is, 0.000 02 for CHP212 +SHSY5Y vs SKNBE+SKNSH, 0.000 03 for CHP212+SKNSH vs SKNBE2+SHSY5Y, and 0.001 44 for CHP212+SKNBE2 vs SHSY5Y+SKNSH. We interpret this first result as a sort of negative control, i.e., the capability of the system as a whole in recognizing subtypes expected to be similar from dissimilar phenotypes. Indeed, this grouping is also suggested by the genomic and phenotypic features, because both CHP212 and SKNBE2 carry 1p36 deletion and *MYCN* amplified while SHSY5Y and SKNSH cells are both wild-type for the same genomic alterations, and moreover, SHSY5Y is a subclone of SKNSH. Following the clues provided by the first-cut data inspection, we designed the hierarchical classifier sketched in Fig. 2(i). It is made of a cascade of three levels of classification, having single classifiers in the first two levels and two alternative classifiers in the last one [L1, L2, L3.1, and L3.2 in Fig. 2(i)]. In the first level L1, the NB cells are identified with respect to the monocytes. Once a NB cell is found, the second level L2 is accessed, in which two intermediate NB classes are discriminated, namely, NB1 (i.e., CHP212 and SKNBE2) and NB2 (i.e., SHSY5Y and SKNSH), consisting of cells lines herein artificially grouped inspiring by the Fig. 2(h). Finally, we access the third level L3 in which, if the NB1 class is detected, the classification L3.1 between the CHP212 and SKNBE2 cells is performed, whereas upon detection of NB2, the classification L3.2 is realized between the SHSY5Y and SKNSH cells. In order to cope with these classification tasks, we created a training set and a test set, as summarized in Table II. In particular, the training set for the classification L1 is made of 10 000 monocytes QPMs and 10 000 NB QPMs. The latter are used to create the training set for the classification L2, i.e., 5000 NB1 QPMs and 5000 NB2 QPMs.

**TABLE II.** Dataset used for training and testing the different levels of the hierarchical classifier.

Cell line		Training set		Test set	
		No. of Cells	No. of QPMs	No. of Cells	No. of QPMs
Monocyte	THP-1	200	10 000	47	4032
Neuroblastoma	CHP212	50	2500	65	5826
	SKNBE2	50	2500	56	5880
	SHSY5Y	50	2500	16	1717
	SKNSH	50	2500	101	12 396
Total		400	20 000	285	29 851

Finally, training sets for the classifications L3.1 and L3.2 are made of 2500 CHP212 QPMs and 2500 SKNBE2 QPMs, and 2500 SHSY5Y QPMs and 2500 SKNSH QPMs, respectively. To avoid data redundancy, we have randomly selected 50 QPMs per cell for each cell lines as highlighted in Table II. The remaining QPMs of the cells belonging to the training set have been discarded and not added to the test set, in order to avoid any bias in the evaluation of the classification performances. Therefore, the test set is created using 29 851 QPMs of cells not used for the training.

**FIG. 3.**
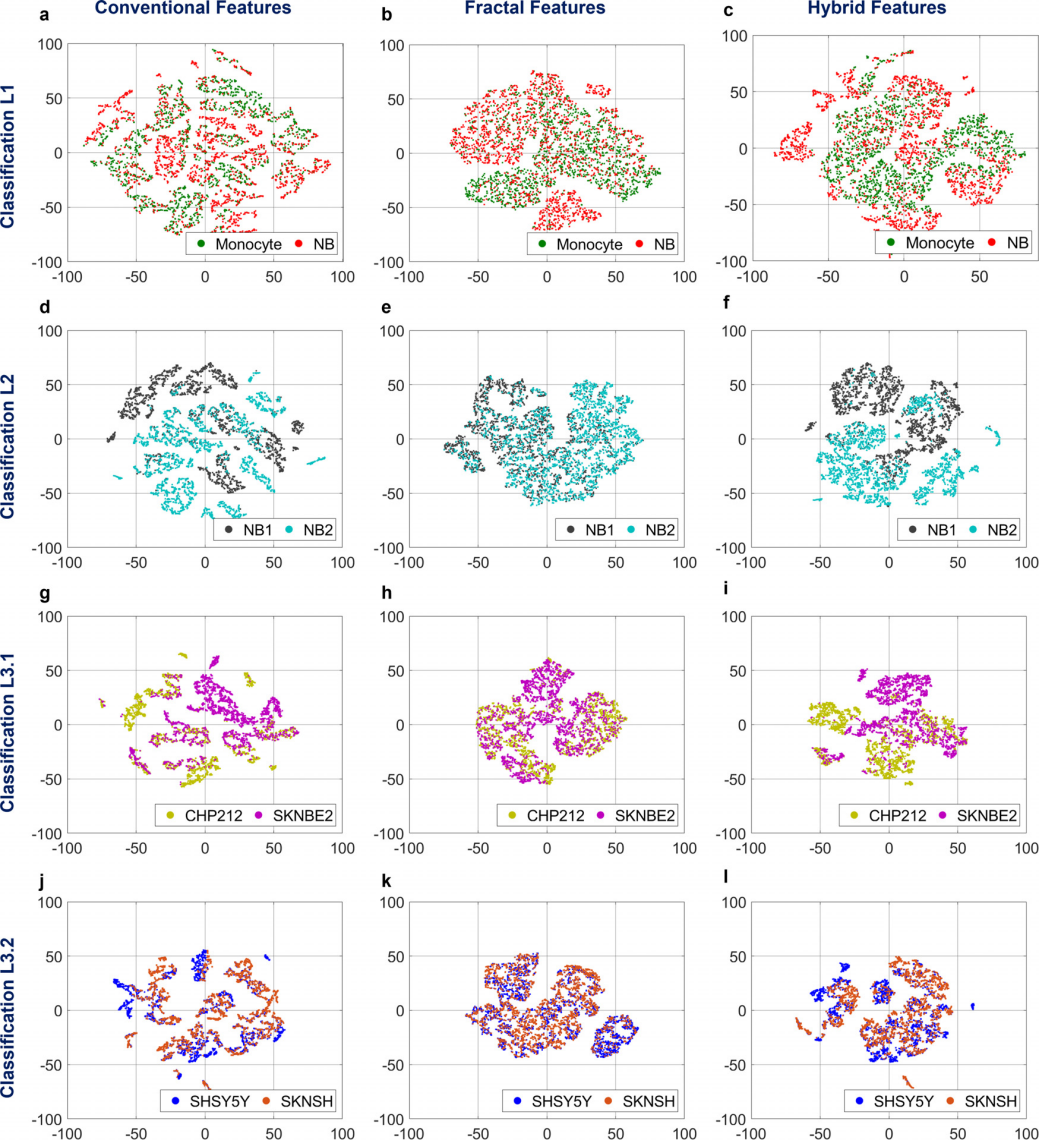
Representation of the training sets by means of the t-SNE algorithm for each classification problem (rows) and for each feature set (columns**)**. (a)–(c) Training set for discriminating monocyte vs NB cells by means of 24 conventional features, 13 fractal features, and 37 hybrid features, respectively. (d)–(f) Training set for discriminating NB1 vs NB2 by means of 24 conventional features, 13 fractal features, and 37 hybrid features, respectively. (g)–(i) Training set for discriminating CHP212 vs SKNBE2 by means of 24 conventional features, 13 fractal features, and 37 hybrid features, respectively. (j)–(l) Training set for discriminating SHSY5Y vs SKNSH by means of 24 conventional features, 13 fractal features, and 37 hybrid features, respectively.

The above training set is further analyzed by using the t-distributed stochastic neighbor embedding (t-SNE) to better inspect the dataset by reducing its dimensionality. In particular, over the rows of Fig. 3, we show the t-SNE results when this is applied to the training sets of the four classifiers by considering the 24 conventional features (first column), the 13 fractal features (second column), and the overall 37 features (third column), termed here hybrid features. Furthermore, for each of the four classification tasks in Fig. 2(i), we computed the Pearson correlation matrix^60^ about all the 37 hybrid features used to train the corresponding classifiers. The results are presented in the supplementary material. the first outcome is that several features are highly correlated among them, as shown in Figs. S1(a), S2(a), S3(a), and S4(a) corresponding to the four classification tasks. However, we averaged the correlation coefficients of the features belonging to the four groups described in Fig. 1(c) (i.e., the OPL-based features, the morphology-based features, the GLCM-based features, and the fractal features), thus obtaining the grouped correlation matrices reported in Figs. S1(b), S2(b), S3(b), and S4(b) for each of the four classifiers, respectively. Here, it can be inferred that the four groups of features are not correlated among them, thus justifying the employment of features with different origins to solve the proposed classification issues. Finally, to understand the impact of different features, the Relief algorithm^61^ has been implemented over the less correlated features (i.e., the features with correlation coefficient lower than 0.9). The rank importance about the selected features is displayed in Figs. S1(c), S2(c), S3(c), and S4(c) for each of the four classification problems. The most distinctive features are the eccentricity in classifications L1 and L3.2 and the area in classifications L2 and L3.1.

### Enhancement of the classification performance through max-voting

**FIG. 4.**
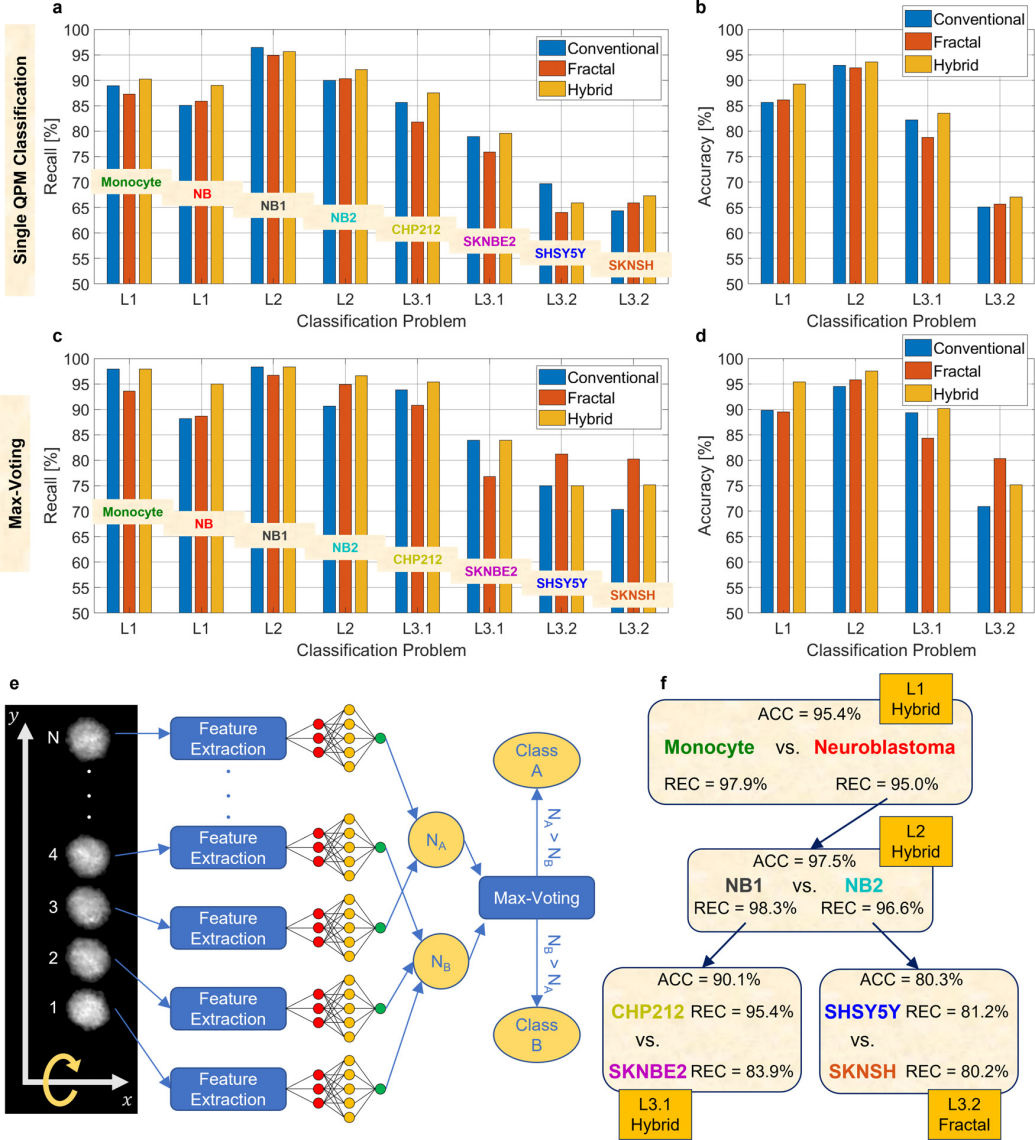
Classification performances within the hierarchical model. (a) and (b) Recall and accuracy, respectively, computed over the QPMs of the test set without max-voting by using the conventional features, fractal features, and hybrid features. (c) and (d) Recall and accuracy, respectively, computed over the cells of the test set through max-voting by using the conventional features, fractal features, and hybrid features. (e) Sketch of the max-voting strategy. For each cell flowing along the y-axis and rotating outside the image plane, N QPMs are recorded. For each QPM, its features are extracted to feed a shallow neural network and predict its class (A or B). The cell is assigned to the class that has occurred more times (N_A > N_B or N_B > N_A). (f) Sketch of the hierarchical model along with the best performances of each classifier obtained after using the reported feature sets combined to max-voting.

The holographic processing, the feature extraction, and the machine learning problems have been solved in Matlab^®^ 2021b environment over an Intel^®^ Core™ i9–9900K CPU with a 64 Gb RAM. In particular, each binary classification task has been solved by training a shallow neural network through conventional features, fractal features, and hybrid features. This classifier has been chosen among the others within the Classification Learner app in Matlab^®^ 2021b (namely, wide neural network) due to its ability of generalizing better to data belonging to the test set never seen before. In particular, it is made of one fully connected layer with 100 nodes and ReLU activation function. The softmax loss has been used as loss function, which is made of a softmax activation followed by a cross-entropy loss. The network's parameters have been initialized through the Glorot method, and the limited memory BFGS algorithm has been used to update the network learnable parameters. The network has been trained by means of 1000 iterations through the Classification Learner app, in which a suitable learning rate is searched automatically. Moreover, a fivefold cross-validation has been used to improve the generalization property. The prediction time of the trained neural network to infer the phenotype of a single QPM is 2.6 ms. To quantify its classification performance, we measure the recall ( REC) and the accuracy ( ACC). Given a binary classification problem between two classes A and B, the recall of class A is defined as

$$\begin{array}{c} {\mathrm{REC}}_{A}=100\frac{T_{A}}{T_{A}+F_{B}} \end{array},$$ (2)

while the accuracy of the classifier is defined as

$$\begin{array}{c} ACC=100\frac{T_{A}+T_{B}}{T_{A}+T_{B}+F_{A}+F_{B}} \end{array},$$ (3)

where $T_{A}$ is the number of elements belonging to class A and correctly classified as class A, $T_{B}$ is the number of elements belonging to class B and correctly classified as class B, $F_{A}$ is the number of elements belonging to class B and wrongly classified as class A, and $F_{B}$ is the number of elements belonging to class A and wrongly classified as class B. Therefore, the recall ${\mathrm{REC}}_{A}$ is the percentage of elements belonging to class A correctly classified as class A, while the accuracy ACC is the overall percentage of elements correctly classified by the model. In Figs. 4(a) and 4(b), respectively, the recall and the accuracy related to the four classification problems reported in Fig. 2(i) have been computed by using the conventional features, the fractal features, and the hybrid features, measured over the QPMs of the test set. As expected, the highest accuracies in Fig. 4(b) are reached with the hybrid features. Moreover, according to what is observed in Fig. 3, the worst accuracy is related to the classification problem L3.2 because of the intrinsic strong similarity between the SHSY5Y and SKNSH NB cells. In this analysis, each QPM of the test set is used separately to predict the class it belongs to. However, it is possible to exploit the fact that multiple QPMs per cell have been collected by implementing a max-voting strategy for each classification task of the hierarchical classifier, thus leading to the performance reported in Figs. 4(c) and 4(d).

The max-voting strategy is sketched in Fig. 4(e) referring to a generic binary classification between classes A and B. On the left, some of the N QPMs of a single cell flowing and rotating along the microfluidic channel are shown. Each QPM is given in input separately to the trained model, thus obtaining $N=N_{A}+N_{B}$ outputs, where $N_{A}$ is the number of QPMs classified as A and $N_{B}$ is the number of QPMs classified as B. Finally, the max-voting strategy consists in assigning a cell to the class most frequently predicted by the trained model, i.e.,

$$\left\{\begin{array}{l} \text{cell}\in A\;if\;N_{A}>N_{B}\\ \text{cell}\in B\;if\;{\;N}_{B}>N_{A}. \end{array}\right.$$ (4)

**FIG. 5.**
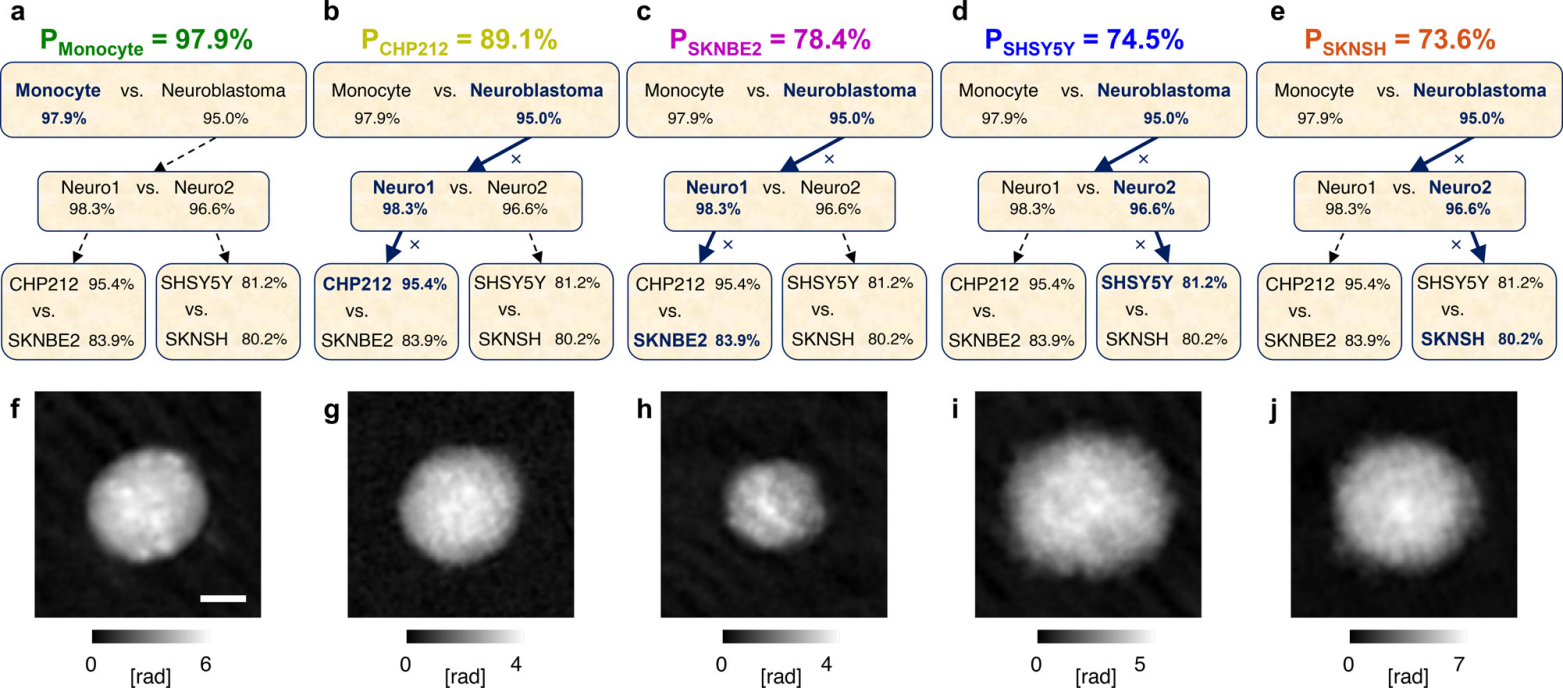
Classification and QPM visualization of several cell lines by means of holographic flow cytometry. (a)–(e) Global probabilities P of correctly classifying monocytes and CHP212, SKNBE2, SHSY5Y, and SKNSH NB cells, respectively, obtained after multiplying the recall values found along each corresponding path inside the best hierarchical tree. (f)–(j) QPMs taken from dataset exploited for the max-voting-based classification of the cell lines in (a)–(e), respectively. Scale bar is 5 *μ*m.

By comparing the results without and with max-voting summarized in Table III and reported in Figs. 4(a) and 4(b) and 4(c) and 4(d), respectively, the enhancement brought by this strategy is evident in terms of performance for each of the classification tasks within the hierarchical model. In particular, by using the max-voting approach for classifiers trained with hybrid features, the accuracy in discriminating monocytes and NB cells (classification problem L1) increases from 89.2% to 95.4%, the accuracy in discriminating NB1 and NB2 cells (classification problem L2) increases from 93.6% to 97.5%, and the accuracy in discriminating CHP212 and SKNBE2 cells (classification problem L3.1) increases from 83.5% to 90.1%. An even more remarkable increment is observed in the most difficult classification problem L3.2, consisting in discriminating SHSY5Y and SKNSH cells, in which the best accuracy obtained without max-voting (i.e., 67.1% by means of the hybrid features) grows up to 80.3%. However, unlike the other cases, the best accuracy is reached by the use of the sole fractal features that outperform the hybrid set. This can be an interesting outcome from the AI perspective, since the sole fractal geometry can handle the huge informative power of a QPM and seems to be able of defining a so distinctive fingerprint for the analyzed cells that even adding the conventional features to the fractal set ends up with a performance worsening. Finally, the best performance obtained by max-voting for each classifier within the hierarchical model is reported in Fig. 4(f). Notice that the recall values reported in Fig. 4(f) are not the probabilities of correctly classifying each cell line, since they refer separately to each single classifier of the hierarchical model. Instead, to obtain the global scores, the probabilities along the several paths of the hierarchical tree must be multiplied. For example, the global probability $P_{\mathrm{CHP}212}$ of correctly classifying a CHP212 cell depends on the probability of correctly classifying a NB cell in the problem L1 (i.e., 95.0%), the probability of correctly classifying a NB1 cell in the problem L2 (i.e., 98.3%), and the probability of correctly classifying a CHP212 cell in the problem L3.1 (i.e., 95.4%). Therefore, by multiplying them, the global probability $P_{\mathrm{CHP}212}=89.1\%$ is obtained. This concept is visually shown in Figs. 5(a)–5(e) for each of the cell lines under analysis with the aim to provide a global perspective of the expected classification accuracy in the case of cell phenotypes identification in a completely blind case where the whole hierarchical tree has to be crossed. The corresponding global probabilities are summarized in Table IV. Furthermore, we compared the global performance of the proposed hierarchical classifier with those obtained through two other possible solutions, i.e., a nonhierarchical model and a hierarchical model with two levels of classification [the intermediate level L2 shown in Fig. 4(f) is avoided], again following the max-voting criterion and trained by using the hybrid features (that yielded the best results). In particular, in the nonhierarchical model, the shallow neural network is trained to directly solve a five-class classification problem. Instead, the hierarchical model with two levels of classification solves first a binary classification problem between monocytes and NB cells and then a four-class classification problem among all the NB subtypes. However, as reported in Table IV, the performance of the nonhierarchical and hierarchical models with two levels of classification are lower than the hierarchical model with three levels of classification proposed here, which strongly supports the adopted approach.

**TABLE III.** Performances of the trained classifiers evaluated over the single QPMs of the test sets without max-voting and over the single cells of the test sets with max-voting. For each classification problem, the best score is given in bold. Values are expressed in %.

Classification problem	Feature set	Recall (%)				Accuracy (%)	
		No max-voting (QPMs)		Max-voting (cells)		No max-voting (QPMs)	Max-voting (cells)
L1		Monocyte	NB	Monocyte	NB		
	Conventional	88.9	85.1	97.9	88.2	85.6	89.8
	Fractal	87.3	85.9	93.6	88.7	86.1	89.5
	Hybrid	90.2	89.0	**97.9**	**95.0**	89.2	**95.4**
L2		*NB1*	*NB2*	*NB1*	*NB2*		
	Conventional	96.4	90.0	98.3	90.6	92.9	94.5
	Fractal	94.9	90.3	96.7	94.9	92.4	95.8
	Hybrid	95.6	92.1	**98.3**	**96.6**	93.6	**97.5**
L3.1		CHP212	SKNBE2	CHP212	SKNBE2		
	Conventional	85.6	78.9	93.8	83.9	82.2	89.3
	Fractal	81.8	75.9	90.8	76.8	78.8	84.3
	Hybrid	87.5	79.6	**95.4**	**83.9**	83.5	**90.1**
L3.2		SHSY5Y	SKNSH	SHSY5Y	SKNSH		
	Conventional	69.7	64.4	75.0	70.3	65.1	70.9
	Fractal	64.0	65.9	**81.2**	**80.2**	65.7	**80.3**
	Hybrid	65.9	67.3	75.0	75.2	67.1	75.2

**TABLE IV.** Probability of correctly classifying a cell line by means of three different models combined to the max-voting strategy. For each cell line, the best score is given in bold. Values are expressed in %.

Classification model	Cell line				
	Monocyte	CHP212	SKNBE2	SHSY5Y	SKNSH
Hierarchical (3 classification levels)	**97.9**	**89.1**	**78.4**	**74.5**	**73.6**
Hierarchical (2 classification levels)	97.9	80.4	78.0	71.3	60.2
Nonhierarchical	78.7	83.1	69.6	56.2	73.3

In summary, we achieve 97.9% probability of correctly identifying a monocyte, 89.1% probability of correctly identifying a CHP212 cell, 78.4% probability of correctly identifying an SKNBE2 cell, 74.5% probability of correctly identifying an SHSY5Y cell, and 73.6% probability of correctly identifying an SKNSH cell. Despite the depth of the hierarchical model has been increased, adding an artificial intermediate level has proved to be the best solution. Moreover, an enhancement of the performance has been possible, thanks to the application of the fractal geometry theory for extracting distinctive cell features and thanks to the max-voting strategy allowed by the holographic flow cytometer used for collecting the data. Hence, the holographic imaging flow cytometry stands as a powerful tool for the cell classification and its downstream analysis based on the dense biophysical information contained in the label-free QPMs. In Figs. 5(f)–5(j), a QPM for each of the analyzed cell line is displayed.

## DISCUSSION

The development of novel technologies for CTCs detection has helped to deepen investigation into the biology of cancer cells and has facilitated their clinical application. For instance, many works have shown the relevance of CTC measurements to monitor treatment response and for evaluating the prognosis of breast, pancreatic and lung cancer patients.^62^ The detection of CTCs is usually dependent on molecular markers, with adhesion molecules of epithelial cells (EpCAM) being the most widely used, although molecular markers vary between different types of cancer. However, these technologies present several limitations, most important being they cannot be used in tumors that are EpCAM-negative or -low. Furthermore, the samples may be contaminated by non-CTCs, such as tumor-associated fibroblasts and endothelial clusters, which increases the false positivity risk.^62^ Usually, these technologies are not designed to recover CTCs for downstream analysis such as RNA or DNA sequencing.

Here, we setup a label-free approach, based on AI and holographic imaging flow cytometry, for rapid and efficient screening and phenotyping of NB cells in the presence of monocytes background. In fact, the implementation of QPI microscopy in combination with flow-cytometry modality allowed us to collect rapidly huge amount of measurement data to ensure the successful application of AI for the problem under consideration. The achieved results represent a solution to the main issue in LB that is the discrimination of anomalous cells into the blood stream, i.e., CTCs, by assessing a method to distinguish between tumor cell and monocytes that are the more similar ones by a morphological point of view.

Furthermore, we can discriminate among four NB cellular types, those with clinically unfavorable genomic aberrations. For the two cell lines of the same origin, our method strongly supported their similarities, but still discriminated the two entities classifying them with high accuracy. These results suggest that the proposed technology could be applied not only to detect CTCs but also to distinguish clinically aggressive from more favorable types of cancers. In NB, a universal and specific cell-surface marker for cells is currently unavailable, even the expression of GD-2 on NB cells varies among studies. To overcome this issue, a recent work has used an epithelial marker-independent enrichment method combined with immunostaining-fluorescence *in situ* hybridization (i-FISH) to detect CTCs from NB patients.^21^ This method utilizes the characterization of centromere of chromosome 8 probe (CEP8) to detect CTCs with hyperdiploidy of chromosome 8 but not all NBs harbor this molecular alteration, and thus, the risk of false negative remains high. One of the advantages of our label-free microscopy-based strategy is that, if isolated by an effective microfluidic chip, CTCs could be used for downstream analysis, such as genomics, transcriptomics, proteomics, and CTCs culture.

While the role of CTCs as biomarkers for diagnosis, prognosis, and therapy monitoring in different cancers has been largely demonstrated, their clinical utility in cancer detection or, in early cancer diagnosis, is still controversial. CTCs are considered a surrogate marker of metastatic activity, but whether cancer intravasation and dissemination of CTCs in patients' blood circulation occurs early during tumor development is still a matter of debate. However, in mouse models, early dissemination seeding metastasis has been identified in breast^63,64^ and pancreatic^65^ carcinogenesis, indicating that CTC circulation is likely to be a very early event in cancer progression. Our preliminary results reported here about the identification of NB cancer cells among WBCs and their successive phenotyping in flow-cytometry modality represent the basis to develop a CTC detection system able to intercept the onset of the tumor in its early stages of development, thus paving the way, in the next clinical practice, for a stain-free liquid biopsy to examine CTC cells for this type of pediatric cancers. Future experiments will be devoted to increase the collected dataset in order to train deep neural networks fed directly by the QPM images instead of the proposed shallow neural network trained as machine learning model through handcrafted features extracted from the recorded QPMs. Indeed, we expect that deep learning would allow increasing the range of detectable NB subtypes and enhancing the classification accuracy about the proposed biomedical issue, above all in the most difficult classification tasks (e.g., L3.2 in the hierarchical classifier herein presented). Moreover, a strategy for enhancing the robustness of the proposed approach could be a multicentric study for the creation of a very large dataset made of holographic images collected in flow cytometry conditions by different laboratories. In this way, the measurement data variations could be better considered during the training step of the proposed classification strategy, thus improving its generalization ability against the different experimental conditions due to the limited possibilities for standardization. A further study will be focused on the analysis of mixed samples at the aim to assess the whole sensitivity of the technology in detecting the percentages of different cell populations, thus mimicking more realistic samples where multiple cell types are present at different concentrations. Indeed, it is worth noting that one limitation of the study is that primary tumor cells have higher variability and diversity than established NB cell lines. However, the latter have been demonstrated to be an important tool to obtain significant advances in cancer research.^66,67^ To confirm that our label-free approach is able to detect CTCs and to distinguish their different tumor phenotypes, we have planned to apply this method to spiked samples (NB cell lines mixed with blood at different concentrations) and then directly to patient blood. We expect that our system would be sufficiently sensitive to detect different types of NB cells in liquid biopsies.

## METHODS

### Sample preparation

The human CHP212, SKNBE2, SHSY5Y, and SKNSH cell lines were obtained from the American Type Culture Collection (ATCC #CRL-2273, #CRL-2271, #CRL-2266, and #HTB-11, respectively). CHP212 cells were grown in Minimal Essential Eagle Medium (MEM; Sigma)/Nutrient Mixture F-12 (F-12), SKNBE2 in Dulbecco's Modified Eagle Medium (DMEM; Sigma)/F-12, SHSY5Y in DMEM, and SKNSH in MEM at 37 °C, 5% CO_2_ in a humidified atmosphere. The medium was supplemented with 10% heat-inactivated FBS (Sigma), 1 mmol/l L-glutamine, penicillin (100 U/ml), and streptomycin (100 mg/ml; Invitrogen). The cell lines were authenticated and early passage cells were used for all the experiments. THP-1 is a human monocytic cell line, supplied by a third part. THP-1 was cultured in suspension in 75 cm^2^ tissue-culture flasks (Corning, product number 353018). To ensure the highest level of viability, they were grown in RPMI 1640 Medium (Life Technologies, ref 31870–025), supplemented with 10% FBS (Life Technologies 10270), 2 mM L-Glutamine (Lonza, Cat N.: BE17–605E), and 1% Penicillin/Streptomycin (Lonza, Cat N. DE17–602E) and maintained at 37 °C in a humidified atmosphere with 5% CO_2_.

To perform the in-flow experiments, CHP212, SKNBE2, SHSY5Y, and SKNSH cell lines were washed twice with PBS 1× (Life technologies 10010023) and incubated for 7 min with 1.5 ml of 0.05% trypsin–EDTA solution (Sigma, T4049–100ML). Then, the cells were resuspended in a PBS solution with 10% FBS, to neutralize the trypsin effect. Instead, THP-1 cells were harvested from the cell culture flask, and spin at approximately 125 × g for 5 min. Then, they were resuspended with the same solution of PBS and 10% FBS. The viability was assessed through Trypan Blue solution 0.4% (Sigma T8154) according to the datasheet. Then, the cells were injected into the microfluidic channel at final concentration of 4 × 100 cells/ml.

### Holographic imaging flow cytometer and numerical processing

For the digital recording of holograms, we employed an off-axis DH setup, based on a Mach–Zehnder optical interferometer. This arrangement grants an angle between object and reference beams and decouples spatially the different diffraction orders in the Fourier space. In particular, as depicted in Fig. 1(a), a solid-state continuous wave laser source (Laser Quantum Torus 532) of wavelength λ=532 nm emits the light beam which is split in object and reference beam by a polarizing beam splitter (PBS). In order to adjust the splitting ratio of the two beams, two half-wave plates (HWPs) are placed in front of and behind the PBS. The object beam passes through the plane of the microfluidic channel, where cells flow and rotate and the light weakly scattered by the sample is collected by a microscope objective (MO_1_ – Zeiss, oil immersion − 40× magnification, 1.3 NA) and sent to a tube lens (TL_1_). The reference beam follows a free path where passes through another MO (MO_2_) and a tube lens (TL_2_). At the end of their paths, object and reference beams are combined by a beam splitter cube (BS) and their interference results in hologram formation digitally recorded by a CMOS camera. The CMOS camera employed is a well-performing camera (Genie Nano-CXP Camera), which consists of an array of 5120×5120 pixels, whose pixel size is Δx=Δy=4.5 μm. The interferometric optical setup provides a magnification of M=36, and thus, a FOV equal to $640\times 640\;\mu m^{2}$ is observable with a lateral resolution of 0.5 μm. The experiment is performed in a microfluidic environment in order to record different viewing angles for each cell in flow. The uniform rotation is grant by a technologically advanced system composed of a low pressure pump module (Cetoni NEMESYS 290 N). It provides a very careful flow rate around 75 nl/s. This flow rate allows a rotation without deformations of the flowing cells inside a commercial microfluidic channel (Microfluidic—ChipShop 10000107). Thus, combining the large FOV and the high throughput provided by the microfluidic environment, our system is able to record hundreds of rotating cells in few minutes. Each in-flow experiment was carried out by inserting into the microfluidic circuit one single type of cell population at a time. A microfluidic protocol has been implemented to avoid contamination between different cell populations by means of several washing steps of the whole circuit (syringe pump, tubes, and microchannel). Such methodology allows accurate and reliable labeling of the cell images. The experiments are performed at room temperature.

For each cell flowing and rotating along the microfluidic channel, hundreds of holograms are recorded, as shown in Fig. 1(b). For each holographic frame, a region of interest (ROI) is selected around the cell. The hologram is first demodulated by filtering the real diffraction order within the Fourier spectrum thanks to the off-axis configuration.^26^ The complex wavefront is then propagated along the optical z-axis through the Angular Spectrum formula^26^ in order to compute the in-focus distance by minimizing a contrast-based metric (i.e., the Tamura Coefficient).^33^ The argument of the refocused complex field is the wrapped phase map, from which the residual phase aberrations are removed by means of a reference hologram,^26^ i.e., a hologram without samples in the imaged FOV. After denoising the wrapped phase map,^68^ an unwrapping algorithm^69^ is implemented to obtain the QPM, as displayed in Figs. 5(f)–5(j).

The holographic experiments to record the dataset in Table I took about 3 h (about 1 h for monocytes and about 30 min for each NB subtype). For each holographic ROI, the numerical processing for reconstructing the corresponding QPM takes 7.71 s, which can be reduced down to 0.17 s by means of deep learning.^36^

### Feature extraction

**FIG. 6.**
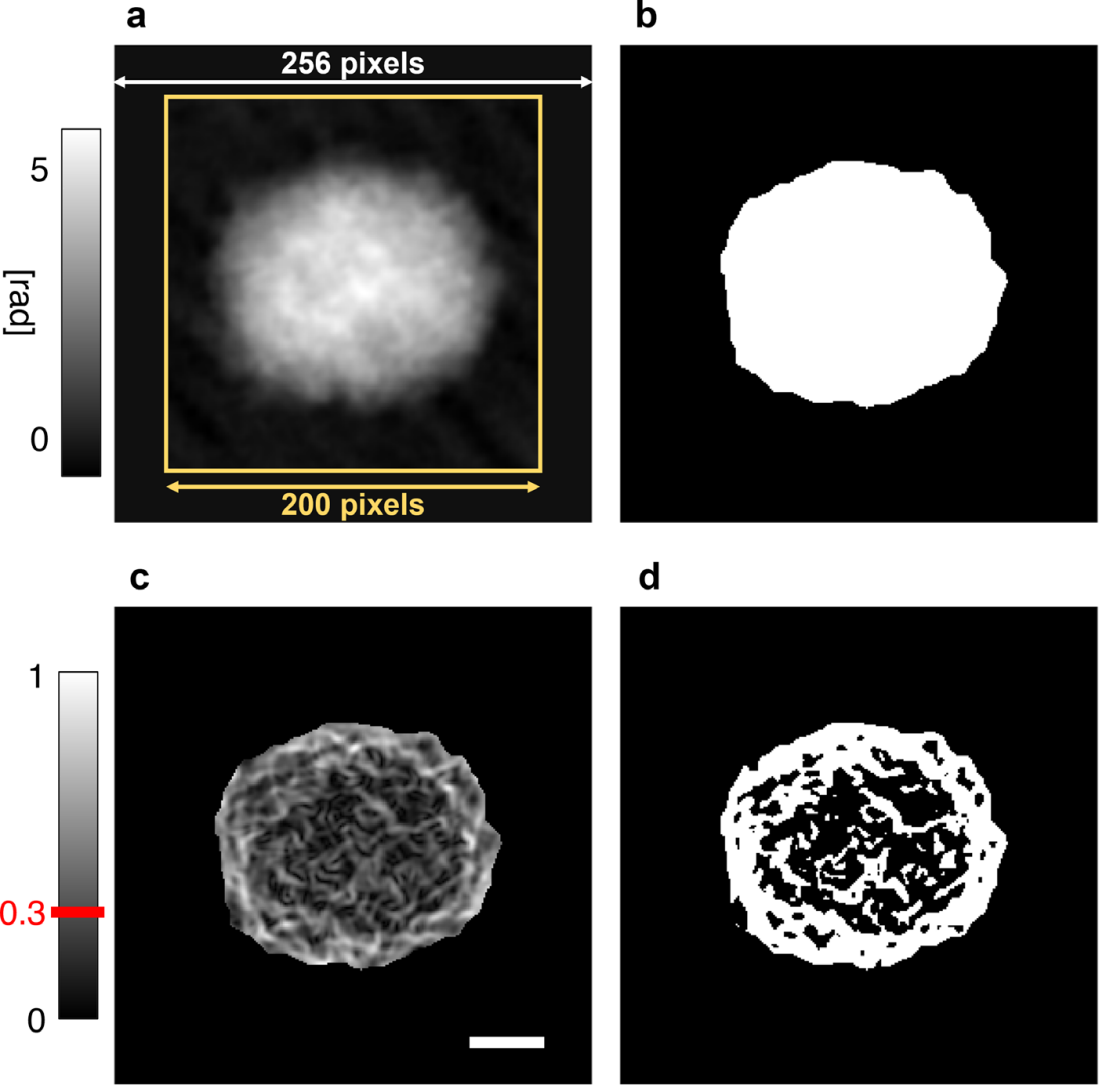
2D images used for feature extraction. (a) QPM ( 200×200 square pixels) of an SHSY5Y cell (yellow) and its zero-padded 256×256 version. (b) Support map obtained by segmenting the zero-padded QPM in (a). (c) Gradient magnitude of the zero-padded QPM in (a) normalized to its maximum value. (d) Hole support map obtained by applying a 0.3 thresholding to the normalized gradient magnitude in (c). Scale bar is 5 *μ*m.

In order to characterize the QPM dataset, 37 hybrid features have been computed for each QPM, divided into 24 conventional features and 13 fractal features, as summarized in Fig. 1(c). As regards the 24 conventional features, they are in turn made of 11 OPL-based features, 9 morphology-based features, and 4 GLCM-based features. The OPL-based features are computed from the phase values of the cell segmented in its QPM [see Figs. 6(a) and 6(b)]. In particular, they include the mean value, the standard deviation, the maximum value, the skewness, the entropy, the kurtosis, the median, the I quartile, the III quartile, and the mode of the cell phase values. In addition, to measure the amount of non-aqueous content inside the cell,^27^ the dry mass is computed as

$$\begin{array}{c} m=\frac{\lambda }{2\pi \gamma }\iint_{x,y}^{}\mathrm{QPM}\left(x,y\right)d\text{xdy} \end{array},$$ (5)

where λ=532 nm is the central wavelength and γ=0.2 ml/g is the refractive increment.^70^ Instead, the morphological features are based on the 2D cell morphology, thus including the cell area, the extent (i.e., the ratio between the cell area and the area of the bounding box, that is the smallest box containing the segmented cell), the solidity (i.e., the ratio between the cell area and the area of the convex hull that encloses the cell), and the maximum diameter and minimum diameter (i.e., respectively the maximum and minimum distance between any two boundary points on the antipodal vertices of convex hull that encloses the segmented cell). Circularity is obtained as

$$\begin{array}{c} C=\frac{4\pi A}{P^{2}} \end{array},$$ (6)

where A and P are the cell area and perimeter, respectively. Moreover, let the equivalent ellipse be the ellipse having the same second-order moments as the segmented cell. The major axis is the length of the major axis of the equivalent ellipse, while the eccentricity is the ratio of the distance between the foci of the equivalent ellipse and its major axis length. Finally, normalized centroids distance is the distance between the centroid and the weighted centroid of the segmented cell, normalized to its equivalent radius, which is the radius of a circle having the same area of the segmented cell. The GLCM-based features are then obtained from the GLCM of the segmented cell. The GLCM takes into account the different combinations of the gray levels within an image. Indeed, the GLCM $G\left(i,j,\theta ,d\right)$ measures how many times a pixel with value i occurs along the direction θ at distance d in respect to a pixel with value j. Herein, among the different GLCMs depending on the values of the offset d and the angle θ, we have chosen the parameters d=1 and θ=0°, and we have measured its contrast, correlation, energy, and homogeneity.^71^

As regards the characterization of the QPMs based on fractal geometry, we computed the 13 fractal features defined in Ref. 55, i.e., the fractal dimension, lacunarity index, fill ratio, regularity index, vertex density, vertex lacunarity index, vertex regularity index, fractal dimension contrast, lacunarity contrast, vertex lacunarity contrast, fractal dimension RMSE, lacunarity RMSE, and vertex lacunarity RMSE. At this aim, as shown in Fig. 6(a), the QPM has been zero-padded in order to pass from a 200×200 square pixels size to a 256×256 square pixels size, since the numerical implementation of the fractal geometry principles requests a power of 2 size. Then, from the zero-padded QPM, two auxiliary maps have been calculated, i.e., the support map and the hole support map. The support map corresponds to the binary map with the segmented cell obtained from the padded QPM, as displayed in Fig. 6(b).

Instead, to obtain the hole support map, the gradient magnitude is computed from the padded QPM and normalized to its maximum value [see Fig. 6(c)], and then a 0.3 threshold is applied, thus leading to the binary map in Fig. 6(d). The support map and the hole support map are finally exploited to compute the 13 fractal features.^57^

The calculation of all the 37 features about a single QPM was obtained in 1.6 s.

## SUPPLEMENTARY MATERIAL

See the supplementary material for the details about the contribution of each feature to the four classification tasks.

## Data Availability

The data that support the findings of this study are available from the corresponding authors upon reasonable request.

## References

[1] K. K. Matthay , J. M. Maris , G. Schleiermacher , A. Nakagawara , C. L. Mackall , L. Diller , and W. A. Weiss , Nat. Rev. Dis. Primers 2, 16078 (2016). 10.1038/nrdp.2016.78 27830764

[2] M. Capasso and S. J. Diskin , Cancer Genet. 155, 65–84 (2010). 10.1007/978-1-4419-6033-7 20517688

[3] X. He , C. Qin , Y. Zhao , L. Zou , H. Zhao , and C. Cheng , Cancer Commun. 40(2–3), 105–118 (2020). 10.1002/cac2.12016 PMC7163660 32237073

[4] M. R. Esposito , A. Binatti , M. Pantile , A. Coppe , K. Mazzocco , L. Longo , M. Capasso , V. A. Lasorsa , R. Luksch , S. Bortoluzzi , and G. P. Tonini , Int. J. Cancer 143(10), 2525–2536 (2018). 10.1002/ijc.31748 29992558

[5] D. Formicola , G. Petrosino , V. A. Lasorsa , P. Pignataro , F. Cimmino , S. Vetrella , L. Longo , G. P. Tonini , A. Oberthuer , A. Iolascon , M. Fischer , and M. Capasso , J. Transl. Med. 14(1), 142 (2016). 10.1186/s12967-016-0896-7 27188717 PMC4870777

[6] G. P. Tonini and M. Capasso , Cancer Metastasis Rev. 39(1), 275–285 (2020). 10.1007/s10555-020-09843-4 31927719

[7] M. Peifer , F. Hertwig , F. Roels , D. Dreidax , M. Gartlgruber , R. Menon , A. Krämer , J. L. Roncaioli , F. Sand , J. M. Heuckmann , F. Ikram , R. Schmidt , S. Ackermann , A. Engesser , Y. Kahlert , W. Vogel , J. Altmüller , P. Nürnberg , J. Thierry-Mieg , D. Thierry-Mieg , A. Mariappan , S. Heynck , E. Mariotti , K. Henrich , and M. Fischer , Nature 526(7575), 700–704 (2015). 10.1038/nature14980 26466568 PMC4881306

[8] M. Capasso , V. A. Lasorsa , F. Cimmino , M. Avitabile , S. Cantalupo , A. Montella , B. De Angelis , M. Morini , C. de Torres , A. Castellano , F. Locatelli , and A. Iolascon , Cancer Res. 80(3), 382–393 (2020). 10.1158/0008-5472.CAN-19-2883 31784426

[9] V. A. Lasorsa , A. Montella , S. Cantalupo , M. Tirelli , C. de Torres , S. Aveic , G. P. Tonini , A. Iolascon , and M. Capasso , Cancer Res. 82(7), 1193–1207 (2022). 10.1158/0008-5472.CAN-20-3788 35101866

[10] D. A. Weiser , D. C. West-Szymanski , E. Fraint , S. Weiner , M. A. Rivas , C. W. Zhao , C. He , and M. A. Applebaum , Cancer Metastasis Rev. 38, 553–571 (2019). 10.1007/s10555-019-09825-1 31836951 PMC6995761

[11] G. De Rubis , S. R. Krishnan , and M. Bebawy , Trends Pharmacol. Sci. 40(3), 172–186 (2019). 10.1016/j.tips.2019.01.006 30736982

[12] H. M. Huang and H. X. Li , Cancer Commun. 41, 91–108 (2021). 10.1002/cac2.12129 PMC7896752 33377623

[13] F. Cimmino , V. A. Lasorsa , S. Vetrella , A. Iolascon , and M. Capasso , Front. Oncol. 10, 596191 (2020). 10.3389/fonc.2020.596191 33381456 PMC7769379

[14] V. Plaks , C. D. Koopman , and Z. Werb , Science 341(6151), 1186–1188 (2013). 10.1126/science.1235226 24031008 PMC3842225

[15] K. Pantel and M. R. Speicher , Oncogene 35(10), 1216–1224 (2016). 10.1038/onc.2015.192 26050619

[16] M. Yu , A. Bardia , B. S. Wittner , S. L. Stott , M. E. Smas , D. T. Ting , S. J. Isakoff , J. C. Ciciliano , M. N. Wells , A. M. Shah , K. F. Concannon , M. C. Donaldson , L. V. Sequist , E. Brachtel , D. Sgroi , J. Baselga , S. Ramaswamy , M. Toner , D. A. Haber , and S. Maheswaran , Science 339(6119), 580–584 (2013). 10.1126/science.1228522 23372014 PMC3760262

[17] A. Satelli , A. Mitra , Z. Brownlee , X. Xia , S. Bellister , M. J. Overman , S. Kopetz , L. M. Ellis , Q. Meng , and S. Li , Clin. Cancer Res. 21(4), 899–906 (2015). 10.1158/1078-0432.CCR-14-0894 25516888 PMC4334736

[18] J. Kapeleris , A. Kulasinghe , M. E. Warkiani , C. Oleary , I. Vela , P. Leo , P. Sternes , K. O'Byrne , and C. Punyadeera , Transl. Lung Cancer Res. 9(5), 1795 (2020). 10.21037/tlcr-20-521 33209602 PMC7653113

[19] K. C. Lin , L. L. Ting , C. L. Chang , L. S. Lu , H. L. Lee , F. C. Hsu , J. F. Chiou , P. Y. Wang , T. Burnouf , D. C. Y. Ho , K. C. Yang , C. Y. Chen , C. H. Chen , C. Z. Wu , and Y. J. Chen , Cancers 13(23), 6076 (2021). 10.3390/cancers13236076 34885184 PMC8656523

[20] Z. Habli , W. AlChamaa , R. Saab , H. Kadara , and M. L. Khraiche , Cancers 12(7), 1930 (2020). 10.3390/cancers12071930 32708837 PMC7409125

[21] X. Liu , Z. Zhang , B. Zhang , Y. Zheng , C. Zheng , B. Liu , S. Zheng , K. Dong , and R. Dong , EBioMedicine 35, 244–250 (2018). 10.1016/j.ebiom.2018.08.005 30104180 PMC6154868

[22] S. Merugu , L. Chen , E. Gavens , H. Gabra , M. Brougham , G. Makin , A. Ng , D. Murphy , A. S. Gabriel , M. L. Robinson , J. H. Wright , S. A. Burchill , A. Humphreys , N. Bown , D. Jamieson , and D. A. Tweddle , Clin. Cancer Res. 26(1), 122–134 (2020). 10.1158/1078-0432.CCR-19-0656 31767563

[23] V. K. Lam , T. C. Nguyen , B. M. Chung , G. Nehmetallah , and C. B. Raub , Cytometry, Part A 93(3), 334–345 (2018). 10.1002/cyto.a.23316 PMC8245299 29283496

[24] E. Moen , D. Bannon , T. Kudo , W. Graf , M. Covert , and D. Van Valen , Nat. Methods 16(12), 1233–1246 (2019). 10.1038/s41592-019-0403-1 31133758 PMC8759575

[25] B. Kemper and G. Von Bally , Appl. Opt. 47(4), A52–A61 (2008). 10.1364/AO.47.000A52 18239699

[26] M. K. Kim , SPIE Rev. 1(1), 018005 (2010). 10.1117/6.0000006

[27] Y. Park , C. Depeursinge , and G. Popescu , Nat. Photonics 12, 578–589 (2018). 10.1038/s41566-018-0253-x

[28] P. Y. Liu , L. K. Chin , W. Ser , H. F. Chen , C. M. Hsieh , C. H. Lee , K. B. Sung , T. C. Ayi , P. H. Yap , B. Liedberg , K. Wang , T. Bourouina , and Y. Leprince-Wang , Lab Chip 16(4), 634–644 (2016). 10.1039/C5LC01445J 26732872

[29] P. Memmolo , G. Aprea , V. Bianco , R. Russo , I. Andolfo , M. Mugnano , F. Merola , L. Miccio , A. Iolascon , and P. Ferraro , Biosens. Bioelectron. 201, 113945 (2022). 10.1016/j.bios.2021.113945 35032844

[30] P. Lenz , D. Bettenworth , P. Krausewitz , M. Brückner , S. Ketelhut , G. von Bally , D. Domagk , and B. Kemper , Integr. Biol. 5(3), 624–630 (2013). 10.1039/c2ib20227a 23328993

[31] N. Goswami , Y. R. He , Y. H. Deng , C. Oh , N. Sobh , E. Valera , R. Bashir , N. Ismail , H. Kong , T. H. Nguyen , C. Best-Popescu , and G. Popescu , Light 10(1), 176 (2021). 10.1038/s41377-021-00620-8 PMC8408039 34465726

[32] M. Mugnano , P. Memmolo , L. Miccio , S. Grilli , F. Merola , A. Calabuig , A. Bramanti , E. Mazzon , and P. Ferraro , J. Biophotonics 11(12), e201800099 (2018). 10.1002/jbio.201800099 30079614

[33] P. Memmolo , L. Miccio , M. Paturzo , G. Di Caprio , G. Coppola , P. A. Netti , and P. Ferraro , Adv. Opt. Photonics 7(4), 713–755 (2015). 10.1364/AOP.7.000713

[34] J. Min , B. Yao , V. Trendafilova , S. Ketelhut , L. Kastl , B. Greve , and B. Kemper , J. Biophotonics 12(9), e201900085 (2019). 10.1002/jbio.201900085 31169960

[35] D. Pirone , D. Sirico , M. Mugnano , D. Del Giudice , I. Kurelac , B. Cavina , P. Memmolo , L. Miccio , and P. Ferraro , Biomed. Opt. Express 13(11), 5585–5598 (2022). 10.1364/BOE.460204 36733743 PMC9872869

[36] D. Pirone , D. Sirico , L. Miccio , V. Bianco , M. Mugnano , P. Ferraro , and P. Memmolo , Lab Chip 22(4), 793–804 (2022). 10.1039/D1LC01087E 35076055

[37] L. Xin , W. Xiao , L. Che , J. Liu , L. Miccio , V. Bianco , P. Memmolo , P. Ferraro , X. Li , and F. Pan , ACS Omega 6(46), 31046–31057 (2021). 10.1021/acsomega.1c04204 34841147 PMC8613806

[38] D. Roitshtain , L. Wolbromsky , E. Bal , H. Greenspan , L. L. Satterwhite , and N. T. Shaked , Cytometry, Part A 91(5), 482–493 (2017). 10.1002/cyto.a.23100 28426133

[39] C. X. Chen , H. S. Park , H. Price , and A. Wax , Front. Phys. 9, 759142 (2021). 10.3389/fphy.2021.759142

[40] M. Ugele , M. Weniger , M. Stanzel , M. Bassler , S. W. Krause , O. Friedrich , O. Hayden , and L. Richter , Adv. Sci. 5(12), 1800761 (2018). 10.1002/advs.201800761 PMC6299719 30581697

[41] D. K. Singh , C. C. Ahrens , W. Li , and S. A. Vanapalli , Lab Chip 17(17), 2920–2932 (2017). 10.1039/C7LC00149E 28718848

[42] N. Nissim , M. Dudaie , I. Barnea , and N. T. Shaked , Cytometry, Part A 99(5), 511–523 (2021). 10.1002/cyto.a.24227 32910546

[43] M. D. Priscoli , P. Memmolo , G. Ciaparrone , V. Bianco , F. Merola , L. Miccio , F. Bardozzo , D. Pirone , M. Mugnano , F. Cimmino , M. Capasso , A. Iolascon , P. Ferraro , and R. Tagliaferri , IEEE J. Sel. Top. Quantum Electron. 27(5), 5500309 (2021). 10.1109/JSTQE.2021.3059532

[44] Y. Jo , H. Cho , S. Y. Lee , G. Choi , G. Kim , H. S. Min , and Y. Park , IEEE J. Sel. Top. Quantum Electron. 25(1), 6800914 (2018). 10.1109/JSTQE.2018.2859234

[45] M. Rubin , O. Stein , N. A. Turko , Y. Nygate , D. Roitshtain , L. Karako , T. Barnea , R. Giryes , and N. T. Shaked , Med. Image Anal. 57, 176–185 (2019). 10.1016/j.media.2019.06.014 31325721

[46] Z. Göröcs , M. Tamamitsu , V. Bianco , P. Wolf , S. Roy , K. Shindo , K. Yanny , Y. Wu , H. C. Koydemir , Y. Rivenson , and A. Ozcan , Light 7(1), 66 (2018). 10.1038/s41377-018-0067-0 PMC6143550 30245813

[47] C. Işıl , K. de Haan , Z. Gorocs , H. C. Koydemir , S. Peterman , D. Baum , F. Song , T. Skandakumar , E. Gumustekin , and A. Ozcan , ACS Photonics 8(4), 1232–1242 (2021). 10.1021/acsphotonics.1c00220

[48] F. Merola , P. Memmolo , L. Miccio , R. Savoia , M. Mugnano , A. Fontana , G. D'Ippolito , A. Sardo , A. Iolascon , A. Gambale , and P. Ferraro , Light 6(4), e16241 (2017). 10.1038/lsa.2016.241 PMC6062169 30167240

[49] D. Pirone , P. Memmolo , F. Merola , L. Miccio , M. Mugnano , A. Capozzoli , C. Curcio , A. Liseno , and P. Ferraro , Appl. Opt. 60(4), A277–A284 (2021). 10.1364/AO.404376 33690379

[50] D. Pirone , J. Lim , F. Merola , L. Miccio , M. Mugnano , V. Bianco , F. Cimmino , F. Visconte , A. Montella , M. Capasso , A. Iolascon , P. Memmolo , D. Psaltis , and P. Ferraro , Nat. Photonics 16, 851–859 (2022). 10.1038/s41566-022-01096-7 36451849 PMC7613862

[51] D. Pirone , D. Sirico , L. Miccio , V. Bianco , M. Mugnano , D. del Giudice , G. Pasquinelli , S. Valente , S. Lemma , L. Iommarini , I. Kurelac , P. Memmolo , and P. Ferraro , Opto-Electron. Adv. 6, 220048 (2022). 10.29026/oea.2023.220048

[52] D. Pirone , L. Xin , V. Bianco , L. Miccio , W. Xiao , L. Che , X. Li , P. Memmolo , and P. Ferraro , Sens. Actuators, B 375, 132963 (2023). 10.1016/j.snb.2022.132963

[53] P. Memmolo , D. Pirone , D. G. Sirico , L. Miccio , V. Bianco , A. B. Ayoub , D. Psaltis , and P. Ferraro , Intell. Comput. 2, 0010 (2023). 10.34133/icomputing.0010

[54] D. Pirone , A. Montella , D. G. Sirico , M. Mugnano , M. M. Villone , V. Bianco , L. Miccio , A. M. Porcelli , I. Kurelac , M. Capasso , A. Iolascon , P. L. Maffettone , P. Memmolo , and P. Ferraro , Sci. Rep. 13, 6042 (2023). 10.1038/s41598-023-32110-9 37055398 PMC10101968

[55] B. B. Mandelbrot , *The Fractal Geometry of Nature* ( WH Freeman and Company, San Francisco, 1982).

[56] G. A. Losa , T. F. Nonnenmacher , D. Merlini , and E. R. Weibel , “ Fractals in biology and medicine,” in *Mathematics and Biosciences in Interaction*, edited by G. A. Losa , T. F. Nonnenmacher , D. Merlini , and E. R. Weibel ( Springer, Birkhäuser, 2002), Vol. 3.

[57] V. Bianco , D. Pirone , P. Memmolo , F. Merola , and P. Ferraro , ACS Photonics 8(7), 2148–2157 (2021). 10.1021/acsphotonics.1c00591

[58] L. Miccio , F. Cimmino , I. Kurelac , M. M. Villone , V. Bianco , P. Memmolo , F. Merola , M. Mugnano , M. Capasso , A. Iolascon , P. L. Maffettone , and P. Ferraro , View 1(3), 20200034 (2020). 10.1002/VIW.20200034

[59] W. Malina , IEEE Trans. Pattern Anal. Mach. Intell. PAMI-3, 611–614 (1981). 10.1109/TPAMI.1981.4767154 21868980

[60] J. Benesty , J. Chen , Y. Huang , and I. Cohen , “ Pearson correlation coefficient,” in *Noise Reduction in Speech Processing, Springer Topics in Signal Processing* ( Springer, Berlin, Heidelberg, 2009), Vol. 2.

[61] K. Kira and L. A. Rendell , “ The feature selection problem: Traditional methods and a new algorithm,” in *Proceedings of the Tenth National Conference on Artificial Intelligence* (ACM Digital Library, 1992), pp. 129–134.

[62] D. Lin , L. Shen , M. Luo , K. Zhang , J. Li , Q. Yang , F. Zhu , D. Zhou , S. Zheng , Y. Chen , and J. Zhou , Signal Transduction Targeted Ther. 6(1), 404 (2021). 10.1038/s41392-021-00817-8 PMC8606574 34803167

[63] Y. Hüsemann , J. B. Geigl , F. Schubert , P. Musiani , M. Meyer , E. Burghart , G. Forni , R. Eils , T. Fehm , G. Riethmuller , and C. A. Klein , Cancer Cell 13(1), 58–68 (2008). 10.1016/j.ccr.2007.12.003 18167340

[64] H. Hosseini , M. M. Obradović , M. Hoffmann , K. L. Harper , M. S. Sosa , M. Werner-Klein , L. K. Nanduri , C. Werno , C. Ehrl , M. Maneck , N. Patwary , G. Haunschild , M. Gužvić , C. Reimelt , M. Grauvogl , N. Eichner , F. Weber , A. D. Hartkopf , F.-A. Taran , S. Y. Brucker , T. Fehm , B. Rack , S. Buchholz , R. Spang , G. Meister , J. A. Aguirre-Ghiso , and C. A. Klein , Nature 540(7634), 552–558 (2016). 10.1038/nature20785 27974799 PMC5390864

[65] J. Yamaguchi , T. Kokuryo , Y. Yokoyama , T. Ebata , Y. Ochiai , and M. Nagino , Oncogene 40(12), 2273–2284 (2021). 10.1038/s41388-021-01706-8 33649537

[66] S. Campos Cogo , T. Gradowski Farias da Costa do Nascimento , F. de Almeida Brehm Pinhatti , N. de Franca Junior , B. Santos Rodrigues , L. R. Cavalli , and S. Elifio-Esposito , Exp. Biol. Med. 245(18), 1637–1647 (2020). 10.1177/1535370220949237 PMC7802384 32787463

[67] M. Salvadores , F. Fuster-Tormo , and F. Supek , Sci. Adv. 6(27), eaba1862 (2020). 10.1126/sciadv.aba1862 32937430 PMC7458440

[68] Q. Kemao , Appl. Opt. 43(13), 2695–2702 (2004). 10.1364/AO.43.002695 15130009

[69] J. M. Bioucas-Dias and G. Valadao , IEEE Trans. Image Process. 16(3), 698–709 (2007). 10.1109/TIP.2006.888351 17357730

[70] Y. R. He , S. He , M. E. Kandel , Y. J. Lee , C. Hu , N. Sobh , M. A. Anastasio , and G. Popescu , ACS Photonics 9(4), 1264–1273 (2022). 10.1021/acsphotonics.1c01779 35480491 PMC9026251

[71] P. C. Costa , Z. Guang , P. Ledwig , Z. Zhang , S. Neill , J. J. Olson , and F. E. Robles , Biomed. Opt. Express 12(3), 1621–1634 (2021). 10.1364/BOE.416731 33796377 PMC7984798

